# New spastic paraplegia phenotype associated to mutation of *NFU1*

**DOI:** 10.1186/s13023-015-0237-6

**Published:** 2015-02-08

**Authors:** Davide Tonduti, Imen Dorboz, Apolline Imbard, Abdelhamid Slama, Audrey Boutron, Samia Pichard, Monique Elmaleh, Louis Vallée, Jean François Benoist, Heléne Ogier, Odile Boespflug-Tanguy

**Affiliations:** Paris Diderot University – Sorbonne Paris Cité; Inserm U1141, DHU PROTECT, Robert Debré Hospital, Paris, France; Department of Brain and Behavioral Sciences, Unit of Child Neurology and Psychiatry, University of Pavia, Pavia, Italy; Hormonology and Biochemistry Departement, Robert Debré Hospital, AP-HP, Paris, France; Department of Biochemistry, Bicetre Hospital, AP-HP, Le Kremlin Bicetre, France; Departement of Neuropediatrics and Metabolic Diseases, Robert Debré Hospital, AP-HP, 48, Boulevard Sérurier, 75019 Paris, France; Departement of Pediatric Radiology, Robert Debré Hospital, AP-HP, Paris, France; Department of Neuropediatrics, CHRU, University Lille North, Lille, France

**Keywords:** Irons sulfur clusters, Leukoencephalopathy, Spastic paraplegia, *NFU1*

## Abstract

Recently an early onset lethal encephalopathy has been described in relation to mutations of *NFU1*, one of the genes involved in iron-sulfur cluster metabolism. We report a new *NFU1* mutated patient presenting with a milder phenotype characterized by a later onset, a slowly progressive spastic paraparesis with relapsing-remitting episodes, mild cognitive impairment and a long survival. The early white matter abnormalities observed on MRI was combined with a mixed sensory-motor neuropathy in the third decade. Our case clearly suggests the importance of considering *NFU1* mutation in slowly evolving leukoencephalopathy with high glycine concentration.

## Dear editor,

Mutations of *NFU1*, one of the genes involved in iron-sulfur cluster metabolism have been recently reported in 14 patients with an early onset and rapidly fatal encephalopathy and/or severe pulmonary hypertension [1-4]. The patients reported died before the age of 15 months [1-3] except for one who was still alive, but severely affected, at the age of 2.5 years [4]. The MRI performed only in this last patient shown white matter abnormalities with cystic degeneration in the periventricular region and in corpus callosum [4]. The brain autopsy analysis performed in 7 patients, demonstrated also white matter demyelination, vacuolization and astrogliosis. We report a new NFU1 mutated patient with a significantly milder phenotype than previously reported.

### Clinical data

The patient was the second born child of unrelated healthy parents. Pregnancy and delivery were uneventful. Psychomotor development was initially normal. At the age of 18 months, concomitantly to an intercurrent viral illness, he lost the ability to walk, stand, sit, grip, he partially lost head control and spastic tetraparesis appeared. After this acute episode he partially recovered: one year later he was able to sit again and to stand alone but he never regained the ability to walk. He was first evaluated in our department at 17 years of age. Neurological examination showed a spastic paraparesis with mild cognitive impairment. Electrophysiological studies performed at 18 years of age (BAEP, VEP, ERG, SEP, EMG, NCV, EEG) were normal. Progressive scoliosis required spine surgery at 18 years of age. At the age of 24 years he subacutely developed flaccid paraplegia with superficial and deep hypoesthesia, EMG and NCV revealed the presence of a severe mixed motor-sensory neuropathy on lower limbs. Lipoic acid treatment (200 mg/day) has been introduced at the age of 28, without significant improvement. The patient now aged 30 years remains stable. However, during the last year, he developed an acute oedema of the lower limbs related to severe bladder dysfunctions requiring a urinary catheter.

### Radiological findings

**Figure 1:**
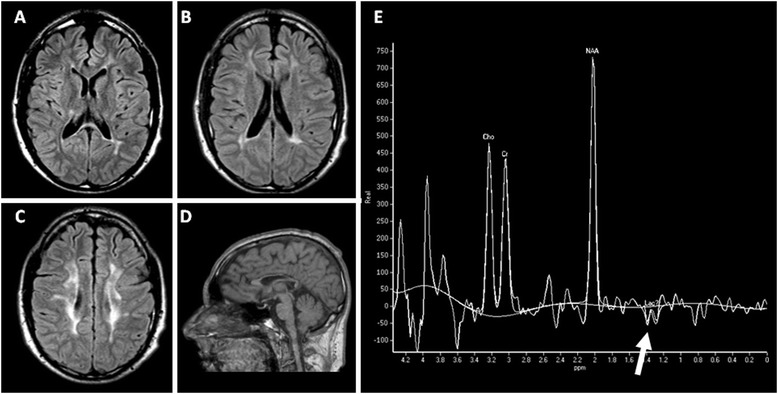
**Radiological findings.** MRI **(A-D)** and H-MRS **(E)** of patient 1 at 28 years of age. FLAIR **(A-C)** and T1 **(D)** sequences. MRI revealed the presence of white matter abnormalities hyperintense on FLAIR sequences, involving the periventricular regions **(A-C)** and the posterior part of corpus callosum **(A)** which was also severely atrophic **(D)**. H-MRS (TE = 136 milliseconds) of the abnormal lobar white matter showed a severe reduction of the N-acetyl aspartate (NAA) peak, an increase of the choline (Cho) peak and a mild doublet inverted peak (white arrow) consistent with elevated lactate (lac).

MRI performed at 3, 7, 16, 19, 24, 28 years of age revealed the presence of white matter abnormalities involving the periventricular regions and the posterior part of the corpus callosum which was also severely atrophic. Single voxel proton MR spectroscopy of the abnormal lobar white matter showed a severe reduction of the N-acetyl aspartate (NAA) peak, an increase in the choline (Cho) peak and a mild double inverted peak consistent with lactate (Figure 1). MRI abnormalities remained globally stable during the long radiological follow-up.

### Laboratory investigations

No documentation concerning laboratory investigations performed before referring to our Institute is still available. Aminoacid chromatographies on urine, plasma and CSF performed at 27 years of age revealed high concentration of glycine (Table 1)

**Table 1:** **Biochemical findings**

**Glycine**	**Plasma (μM)**	**Urines (μM)**	**CSF (μM)**
	**530** (181–293)	**3077** (43–173)	**43** (1–16)
**Redox status**	**Lactate (mM)**	**Pyruvate (mM)**	**Pyruvate/Lactate**
Fasting	1 (0.6-1.9)	94 (50–140)	10, 5 (<10)
Post-prandial	**2.5** (0.6-1.9)	**221** (50–140)	11 (<10)
Mitochondrial enzymatic activities	**Muscle**		**Fibroblasts**
CI	9 (9–13)		NA
CII	**6** (20–42)		**4**, 11-19
CIII	67 (61–132)		60 (33–67)
CIV	**59** (81–171)		51 (41–81)
PDHc	NA		**98** (1372–3104)
CS	**34** (76–140)		42 (31–65)
CIV/CS	1.74 (1.1-1.9)		1.21 (0.9-1.6)
CIV/CII	**9.83** (3.4-6.1)		**12.75** (2.7-5.3)
CIV/CI	**6.56** (10.5-20.9)		NA
CII/CS	**0.18** (0.25-0.41)		**0.10** (0.24-0.38)
AKGDH	5.4 (4–9)		**0.3** (4–9)

Concentration of lactate, pyruvate and glycine in body fluids and mitochondrial enzymatic activities at 27 years of age. Abnormal concentrations are highlighted in bold. Normal values in brackets. CSF = cerebrospinal fluid, CS = citrate synthase, CI = complex I, CII = complex II, CIV = complex IV, PDHc = pyruvate dehydrogenase complex (PDHc) and AKGDH = α-ketoglutarate dehydrogenase.

. Redox status evaluations at 22 and 27 years of age showed a mild elevation of lactic and pyruvic acids after meal (Table 1). Activities of the respiratory chain complexes (mainly complex II) were low in muscle and fibroblasts as well as the pyruvate dehydrogenase complex and the α-ketoglutarate dehydrogenase in fibroblasts (Table 1).

Molecular analysis of *NFU1* revealed the presence of one novel mutation (c.146delC, p.Pro49LeufsX8) predicted to be damaging (Align DGVD, Polyphen-2, SIFT, MutationTaster) inherited from the mother and a previously reported missense mutation (c.565G > A, p.Gly189Arg) [4] inherited from the father.

## Discussion

Iron-sulfur clusters (Fe-S) are essential components of many mitochondrial, nuclear and cytosolic proteins [5]. We described a new patient affected by a disorder of iron-sulfur cluster metabolism due to heterozygous mutations of the *NFU1* gene. All patients so far described presented a clinical picture dominated by an early onset severe encephalopathy and/or a severe pulmonary hypertension that lead in both cases to early death [1-4]. In sharp contrast, our patient presented a milder progressive leucoencephalopathy with a later age of onset (18 months of age) characterized by stress induced deterioration followed by partial recover, as usually observed in mitochondrial diseases [6]. No signs of pulmonary hypertension were ever found. The patient reached an age of 30 years with relatively stable neurological conditions. A subacute demyelinating mixed motor sensory neuropathy, never yet reported in *NFU1* mutations, appeared in the third decade. Peripheral neuropathy is a typical feature in *PDHA1* deficiency [7]. On our patient, *NFU1* mutations result in a severe PDH defect demonstrated in fibroblasts. Moreover, differently to what is reported to date, our patient’s lactate and pyruvate levels were only high postprandially and the lactate/pyruvate ratio was normal. These data suggest a PDH deficiency more than an oxidative phosphorylation deficiency [1-4]. For this reason we suggest the possibility that the neuropathy in this patient may be related to the severe PDH deficiency.

The reason for the clinical and biological differences between our patient and those previously described is not clear. We can observe that patients reported to date were all homozygous either for the same null mutation or for the same missense mutation with only two patient heterozygotes for two different missense mutations [3,4]. Our patient was found to be compound heterozygote for a null and a missense mutation, one never reported so far. We could speculate that this genetic situation and probably some unknown epigenetic factors could differently modulate the final phenotype.

Our case clearly suggests the importance of considering *NFU1* mutation not only in early onset leukoencephalopathy or in presence of a severe pulmonary hypertension but also in a more slowly evolving and later onset leukoencephalopathy with sometimes acute phases of deterioration. High concentration of glycine in body fluids is a key diagnostic marker.

## Abbreviations

BAEP: Brainstem Auditory Evoked Potentials
VEP: Visual Evoked Potentials
ERG: Electroretinogram
SEP: Somatosensorial Evoked Potentials
EMG: Electromyography
NCV: Nerve Conduction Velocity
EEG: Electroencephalogram
MRI: Magnetic Resonance Imaging
NAA: N-Acetyl-Aspartate
CSF: Cerebrospinal fluid
